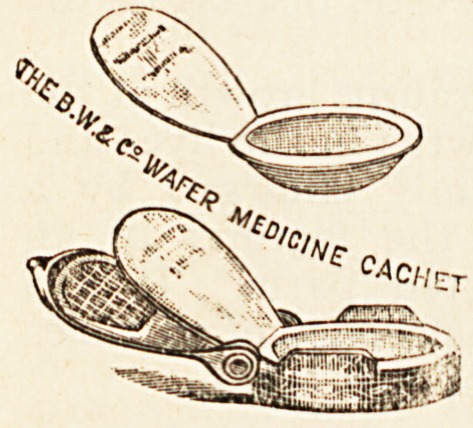# Notes on Preparations for the Sick

**Published:** 1891-03

**Authors:** 


### Notes on Preparations for the Sick.

**Diabetic Foods.**—G. Van Abbott & Sons, London.

The older preparations for diabetics are for the most part well tried, and Messrs. G. Van Abbott & Sons have from time to time added many new kinds of biscuits, cakes, and rusks, made carefully to avoid as much as possible the introduction of sugar or starch. The latest novelty is the use of Soy Bean Flour (from the soya hispida) for the making of bread, biscuits, and rusks. The use of "Soya bread" has recently been advocated for diabetics by no less eminent an authority than Dujardin-Beaumetz. Lucerf, according to Professor Yeo, was one of the first to call attention to the value of the meal of soya hispida in dietetics. The meal is very rich in nitrogenous substance, more so than animal flesh, and the amount of starchy and saccharine substance is very small.

Analysis of the bread gives the following results:—"We find it to contain as much as 25.02 per cent. of nitrogenous matter, and as little as 2.72 per cent. starch, and 4 per cent. of mineral matter. In texture it is just like ordinary whole-meal bread, and it possesses quite an agreeable taste." Messrs. Van Abbott and Sons have evidently made a very valuable addition to their list of foods which are intended for the use of the diabetic. The biscuits are not less satisfactory.

Rigid attention to the question of diet is well known to be an absolute necessity in the graver forms of diabetes, and hence the need for as great a variety as is possible in the list of what a diabetic "*may eat.*"

---

**Fluid Extract of Malt.**—B. Keen, Bristol.

The ordinary thick malt extract is peculiarly nauseous to some patients, and a fluid substitute is likely to be often useful. The fluid extract may be readily combined with wine, milk, and other medicinal fluids, and must be an efficient vehicle for cod-liver oil.

---

**Apioline Chapoteaut. Hamilton's Linseed Leaves. Gouttes Livoniennes.**—Wilcox & Co., London.

The active principle of apium petroselinum, differing from the solid apiol, is dispensed in capsules, each containing 25 centigrammes. Two or three of these prescribed daily at the monthly period, and repeated the following month, have succeeded in bringing on the menses. In cases of dysmenorrhœa and amenorrhœa, we have found the drug to be more efficient than other reputed emmenagogues.

Linseed leaves are used in the same manner as the well-known Rigollot's sinapism: they should be steeped in warm water for one minute, and after application to the part should be covered with gutta-percha tissue, by which means the heat and moisture may be retained for some hours. They appear to be a great improvement on the ancient and now somewhat unpopular linseed poultice.

In **Gouttes Livoniennes** we have a combination of Norwegian tar, beech tar creasote, and balsam of tolu, in the form of capsules. We have found them to be useful in the treatment of phthisis.

**Medicine Cachets.**—Burroughs, Wellcome & Co., London.

These are made of pure rice starch, and are readily soluble by the saliva or the pancreatic ferment of the intestines. They are intended to be employed as a means for disguising the taste of nauseous medicines such as balsam copaiba, cubebs, quinine, and many other medicines where the taste would be objected to. They are also convenient for taking pills or "tabloids" of compressed drugs. When a "tabloid" is enclosed in the rice starch shell it is dipped into a little water, which immediately causes the film to closely fit the contents. When placed on the tongue the cachet does not readily break up, but may be swallowed intact with a draught of water.

These cachets are a very useful addition to the apparatus of the sick-room. Any nauseous powder when prepared in this form is easily swallowed, and is preferable to a pill, inasmuch as it must be much more easily dissolved. We think it should be the duty of the dispenser to prepare the cachets, otherwise mistakes in the dose are likely to occur.

---

**Tonic Port Wine.**—W. & A. Gilbey, London.

This preparation is a wine from which, it is said, tannin and astringent substances have been removed, and in which we have the simultaneous existence of the extracts of the grape, malt and meat. This is a powerful combination, but one which is distinctly medicinal and not altogether agreeable to a sensitive palate. It may be safely given even where other kinds of alcoholic beverages are not desirable.

## Figures and Tables

**Figure f1:**